# Eight challenges in phylodynamic inference

**DOI:** 10.1016/j.epidem.2014.09.001

**Published:** 2015-03

**Authors:** Simon D.W. Frost, Oliver G. Pybus, Julia R. Gog, Cecile Viboud, Sebastian Bonhoeffer, Trevor Bedford

**Affiliations:** a Department of Veterinary Medicine, University of Cambridge, Cambridge, UK; b Institute of Public Health, University of Cambridge, Cambridge, UK; c Department of Zoology, University of Oxford, Oxford, UK; d Department of Applied Mathematics and Theoretical Physics, University of Cambridge, Cambridge, UK; e Fogarty International Center, National Institutes of Health, Bethesda, USA; f Institute of. Integrative Biology, ETH Zurich, Zurich, Switzerland; g Vaccine and Infectious Disease Division, Fred Hutchinson Cancer Research Center, Seattle, USA

**Keywords:** Phylodynamics, Coalescent models, Selection, Recombination

## Abstract

The field of phylodynamics, which attempts to enhance our understanding of infectious disease dynamics using pathogen phylogenies, has made great strides in the past decade. Basic epidemiological and evolutionary models are now well characterized with inferential frameworks in place. However, significant challenges remain in extending phylodynamic inference to more complex systems. These challenges include accounting for evolutionary complexities such as changing mutation rates, selection, reassortment, and recombination, as well as epidemiological complexities such as stochastic population dynamics, host population structure, and different patterns at the within-host and between-host scales. An additional challenge exists in making efficient inferences from an ever increasing corpus of sequence data.

## Highlights

• We outline core challenges in phylodynamic inference.
• Evolutionary challenges include selection and recombination.
• Epidemiological challenges include population structure and stochasticity.
• Methodological challenges include sampling bias and computational complexity.

## Introduction

‘Phylodynamics’ is a term used to describe the ‘melding of immunodynamics, epidemiology, and evolutionary biology’ in order to understand how infectious diseases are transmitted and evolve (Grenfell et al., 2004). Since the term was coined ten years ago, many studies have taken up this concept, driven by the increasing availability of pathogen sequence data. Publicly available software such as BEAST (Drummond and Rambaut, 2007) has enabled individuals to apply complex evolutionary models to these data. New conceptual models (Volz et al., 2009, 2012; Frost and Volz, 2010; Rasmussen et al., 2011; Stadler et al., 2012; Dearlove and Wilson, 2013) have added to our understanding of how the process of disease transmission may shape a phylogeny, and of how the population genetics concept of ‘effective population size’ relates to pathogens. Here we present open challenges in using sequence data to infer disease dynamics.

## 1 How can we account for sequence sampling patterns?

While there are a vast amount of publicly-available sequence data – currently there are over one million viral sequences in GenBank – sampling is often highly biased. Sampling may be biased towards trying to capture a diverse taxonomic sample or may be biased by sampling a restricted geographic area, impacting estimates of effective population size (Holmes et al., 1999). Sampling effects may also be important when studying the ‘phylogeography’ of a pathogen. Some widely used phylogeographic models treat the migration of a pathogen as though it were analogous to mutation (Kühnert et al., 2011), mainly for computational expediency. However, spatial oversampling of specific areas may lead to these areas becoming apparent ‘sinks’, where overrepresentation of a deme causes estimates of migration into that deme to increase.

Previous work on the impact of temporal sampling has demonstrated that sampling protocols designed to capture sequences at specific points in the epidemic cycle result in more accurate inference using coalescent models (Stack et al., 2010). Current birth-death models used for phylodynamic inference assume a constant probability of sampling throughout the evolutionary history, which may result in biased estimates of quantities such as the effective population size when the sampling process is misspecified. Formal investigations of the potentially confounding effects of both spatial and temporal non-random sampling, and how they may be ameliorated, are well overdue. In order to make the best use of currently available data, methods would strongly benefit from recalibrating samples based on surveillance information; the development of realistic models of the sampling process could in theory increase statistical power while reducing bias.

## 2 How can more realistic evolutionary models be used to improve phylodynamic inferences?

For rapidly evolving pathogens, a range of sampling times can be used to calibrate a ‘molecular clock’ to estimate divergence times on a phylogenetic tree. Despite ‘relaxed’ clocks that can capture some degree of variation in evolutionary rates (Drummond et al., 2006), current models may fall short of capturing true variation. For example, Wertheim et al. (2012) analysed major subtypes of pandemic HIV-1 group M, which are thought to exemplify closely related lineages with different substitution rates, and found that the times to the most recent common ancestor differed markedly when subtypes were analysed separately compared to jointly. This suggests that current models fail to capture higher-order temporal correlations in the evolutionary rate. However, recent work on influenza by Worobey et al. (2014) found that incorporating outside information, in this case host species, substantially improved temporal calibration.

In addition, most studies do not consider how the epidemiological dynamics may feed back to the pattern of evolution. For pathogens such as influenza A, mutations that induce antigenic change and allow escape from the predominating herd immunity are likely to spread. This results in phylogenies that depart strongly from the neutral coalescent expectation, showing increased asymmetry in branching topology and skewed patterns of coalescence (Bedford et al., 2011). Analytically tractable coalescent models that directly incorporate such selection pressures do not currently exist. Recent progress has been made applying non-Kingman coalescent processes, such as the Bolthausen–Sznitman coalescent, to capture some of the broad effects of selection on phylogenetic shape and scale (Neher and Hallatschek, 2013). However, even with such coalescent models, there will remain the assumption that the observed phylogeny is independent of the substitution process. This is problematic as a major goal is to link viral mutations to evolutionary outcomes, and identify strains that may have a competitive advantage.

## 3 What is the role of stochastic effects in phylodynamics?

The vast majority of phylodynamic studies assume a time-varying coalescent model (Pybus and Rambaut, 2009; Volz et al., 2013) that specifies that changes at the population level are deterministic, which have demonstrated a variety of dynamic patterns for different viral systems (Table 1 in Frost and Volz (2010)). Demographic stochasticity may play a central role in infections that exhibit recurrent epidemics, such as influenza A virus and norovirus, due to seasonal troughs in incidence. However, even infections that are now endemic in many populations, such as HIV-1 and hepatitis C virus, were once at low frequency, and also sporadically appear in new populations. Hence, stochastic effects may play a role close to the time of the most recent common ancestor for many pathogens. Stochastic effects due to demography may also be important when the number of infected individuals is relatively small and/or infection and recovery rates are high.

Several recent studies have employed a stochastic linear birth–death process, where birth corresponds to transmission, and death to either recovery or death of infected individuals (Stadler et al., 2012). However, these models assume constant rates, and hence may be inappropriate if infection is not spreading exponentially. Extensions to the basic birth–death model, such as the birth–death skyline (Stadler et al., 2013), which involves fitting a piecewise constant birth–death process, may help to capture varying infection rates. However, fitting a stochastic, nonlinear model of disease transmission may be preferable to such nonparametric approaches, as it may offer mechanistic insights. One approach to incorporate stochasticity in such models is to apply the coalescent to an ensemble of stochastic simulations, an approach taken by Rasmussen et al. (2011), who fitted a stochastic differential equation model jointly to epidemiological data and to coalescence events inferred from a phylogenetic tree. Another possible approach to allow use of the coalescent likelihood would be to perform a stochastic change in timescale (Kaj and Krone, 2003). Kühnert et al. (2014) fitted a stochastic epidemiological model by simulating epidemiological trajectories, which were used to parameterise a stochastic birth-death process with piecewise constant rates. One can take inspiration from developments in fitting stochastic epidemic models to incomplete data. Leventhal et al. (2014) fitted a stochastic model using numerical approximation to the solution of the underlying master equation, in order to integrate out the (unknown) number of transmission events in the population that occur between coalescent intervals in the sample.

Environmental stochasticity, which is important in the dynamics of many vector-borne diseases, has received relatively little attention in the phylodynamics literature to date. Recent developments in fitting models that can accommodate both stochasticity (via stochastic differential equations, a common framework for including environmental stochasticity) (Rasmussen et al., 2011) and structure (Rasmussen et al., 2014) are encouraging, and await wider availability of sequence data on vector-borne pathogens.

## 4 How does the structure of the host population relate to pathogen genetic variation?

There has been recent progress in understanding how classical compartmental epidemiological models, which can incorporate population structure by considering multiple classes of individual, relate to the resulting pathogen phylogeny (Stadler et al., 2012; Volz et al., 2012; Volz, 2012; Frost and Volz, 2013). These models may be more robust to biased sampling of specific groups than models that consider population structure as a trait that evolves independently of the underlying phylogeny (Lemey et al., 2009). Including population structure may be essential for accurate inference, as recently demonstrated by Rasmussen et al. (2014a), who showed that a panmictic epidemiological model failed to capture the classical oscillatory dynamics of dengue virus, while splitting the hosts into separate but linked urban and rural populations was sufficient to recapitulate observed oscillations in hospital admissions with dengue. Phylogeographic models have also been applied to consider host species jumps in multiple host systems such as rabies Streicker et al. (2010), although there are issues with sparse sampling of such systems, and the potential for stochastic effects (Buhnerkempe et al., 2014; Lloyd-Smith et al., 2014).

However, compartmental models may not fully capture heterogeneity in contacts and transmissions among individuals. A high variance among hosts in onward transmission – sometimes termed super-spreading – is characteristic of many infectious diseases (Lloyd-Smith et al., 2005), and this may impact the phylogeny (Leventhal et al., 2012). There are many challenges in developing network models that aim to capture deviations from the ‘well-mixed pot’ assumption of many compartmental epidemiological models (Pellis et al., 2014), and more challenging still to include such structure into phylodynamic models. This relates to the wider challenge of incorporating individual-level variation, rather than aggregating individuals into groups.

It may be possible to reverse engineer population structure from the pathogen phylogeny even when the population structure is not directly measured. Stadler and Bonhoeffer (2013) proposed a multitype birth-death type model that can in principle identify superspreaders in a population, by considering multiple groups, with membership as a latent variable. In addition, the structured coalescent approach proposed by Volz et al. (2012) and extended by Rasmussen et al. (2014), which deals with probabilities of being in a specific state, also lends itself to modeling such ‘hidden’ or latent group membership.

## 5 How can we incorporate recombination and reassortment?

Many viruses are known to recombine at a high rate, and failing to take recombination into account when it is present is likely to lead to errors in inference. For example, standard phylogenetic approaches are more likely, under many circumstances, to reconstruct a ‘star-like’ tree when applied to sequence data affected by recombination, which resembles the pattern produced under exponential growth. Incorporating recombination would not only help to avoid such errors, but may also provide further insights into transmission dynamics. Recombination occurs during multiple infection, implying that pathogens – potentially with different times and places of origin – were in the same host at the same time. Unfortunately, the theoretical and computational problems involved in implementing general models of recombination in phylodynamics are significant, and most phylodynamic studies to date assume that there is no recombination, or or employ a ‘multiple loci’ model which assumes free recombination among sequence partitions, but no recombination within them (*e.g.* Lemey et al. (2004)). Progress may be made most readily for viruses with segmented genomes that exhibit reassortment, such as influenza A virus, because in that instance only the frequency of reassortment, and not the breakpoint locations need to be inferred. The problem of jointly modeling phylogenies in different, potentially reassorting segments, is similar to the problem of host-parasite cophylogeny, which tries to reconcile potentially conflicting host and parasite phylogenies to help understand how speciation in the host may affect speciation in the parasite (see *e.g.* Jackson and Charleston (2004)).

Joint inference of phylogeny and recombination patterns, *i.e.* an ancestral recombination graph or ARG, after a long hiatus (Kuhner et al., 2000), is undergoing a renaissance (O’Fallon, 2013; Rasmussen and Siepel, 2013). The development of standards to represent ARGs, such as ArgML (McGill et al., 2013) will help to facilitate development of these methods. However, to truly integrate these models of recombination with mechanistic epidemiological models will require consideration of the many factors that impact recombination (Worobey and Holmes, 1999).

## 6 How can we include phenotypic as well as genotypic information?

While phylodynamic approaches to date have focused on pathogen genotypes, many phenotypes may influence transmission patterns, including replication rate, pathogenicity, host specificity, tropism and antigenicity. Appropriately incorporating such phenotypic information into phylodynamic studies remains an outstanding challenge.

Although there has been progress made towards study of antigenic phenotype by applying “cartographic” models to serological data (Smith et al., 2004; Bedford et al., 2014), there may be other more-appropriate serological models, especially in terms of incorporating the effects of polyclonal sera or cellular immunity. In the context of modeling within-host viral genetic variation, it may be fruitful to consider how the neutralising antibody repertoire co-evolves with the pathogen, a possibility raised by the ability to sequence immunoglobulin genes in a high throughput fashion (Fischer, 2011). Another phenotypic measure of interest is the replication rate of the pathogen, for which high-throughput phenotypic assays of viruses are becoming increasingly available (Kouyos et al., 2012). Although even in the absence of such assays, there may be proxies of replication rate that can be used instead. For example, Alizon et al. (2010) showed that viral ‘setpoint’ in HIV-1 exhibited heritability across the viral phylogeny. In each of these cases, there is significant opportunity to link specific sequence mutations to changes in phenotype, as well as to determine how phenotype influences the pathogen transmission process. Such an approach may provide insights into complex host–pathogen dynamics of acute infections where herd immunity may play a role, such as strain interactions in dengue (Reich et al., 2013) and antigenic seniority in influenza (Lessler et al., 2012). However, determining and measuring various aspects of pathogen fitness, including replication rate and immune recognition, is in itself a significant challenge (Metcalf et al., 2014).

## 7 How can we capture pathogen evolution at both within- and between-host scales?

Most phylodynamic models assume that the timing of coalescent events in the phylogeny coincide with the timing of transmission events, which is not the case when there is significant genetic variation within the infected host. In the past, the absence of sequence data at both the within- *and* between-host scales has limited our ability to address this issue. With the decreasing cost of sequencing, studies of pathogen evolution at multiple scales are now possible. However, with few exceptions (Dearlove and Wilson, 2013), most studies do not permit the combination of data at different scales. This may be a particular concern when applying models to small, well sampled populations, for example, in outbreak situations (Ypma et al., 2013).

Patterns of between-host evolution do not just reflect a rescaling of within-host patterns (Gog et al., 2014), and capturing different mechanisms for evolution at multiple scales within phylodynamic models presents a significant challenge to the field. Phenomena that should be considered include host-specific immune responses, temporal changes in selection pressure during the course of infection, founder effects, biased transmission of specific pathogen variants, and in the case of retroviruses such as HIV-1, the storage of the virus in the body. These processes can contribute to the substantially faster rates of within-host evolution relative to between-host evolution (Lemey et al., 2006; Pybus and Rambaut, 2009).

## 8 How can analytical approaches keep up with advances in sequencing?

Large sample sizes pose significant problems for phylogenetic inference, as the number of possible tree topologies increases double-factorially with the number of taxa, and the largest phylodynamic data sets (for HIV-1 and influenza A virus) consist of many thousands of whole genomes. One solution to reduce the considerable time taken to fit these models is to harness modern graphics processing units (GPUs) to speed up the computation of phylogenetic likelihoods (Suchard and Rambaut, 2009; Ayres et al., 2012). This approach appears particularly beneficial when large Markov rate matrices are used, for example in the calculation of codon substitution models, or when treating alignments with many sites (as opposed to many taxa). However, approaches for computing phylogenetic likelihoods that are also efficient for large numbers of sequences are sorely needed. Progress in the challenges described above are likely to make the situation of long running times even worse. Development of algorithms that can, for example, improve mixing of Markov chain Monte Carlo approaches commonly used in phylodynamic studies, or at least provide rapid, approximate results for exploratory data analysis would further open up possibilities for the analysis of ‘big data’.

## Discussion

The growing resource of sequence data provides the non-trivial challenge of converting genetic information into inferences of infectious disease dynamics. Genetic sequences contain a wealth of information on the transmission process, but rigorously harnessing this information is a difficult task. We have outlined some of the inferential challenges that lie in front of us. These are mainly challenges in going beyond simple compartmental epidemiological models and also in going beyond simple neutral non-recombining evolutionary models. Elsewhere in the issue, challenges in addressing broader evolutionary questions are raised (see ‘Five challenges in evolution and infectious diseases’, Metcalf et al. (2014)), e.g. how can we understand the origins of selective pressure on a virus population, as opposed to our focus on how to deal with selective coefficients? Yet elsewhere, challenges in metapopulation dynamics (‘Challenges for metapopulation models of epidemics’, Ball et al. (2014)) and spatial structure (‘Five challenges for spatial epidemic models’, Riley et al. (2014)) are presented. We believe phylodynamic approaches have particular promise in addressing some of these challenges as they allow more direct observation of mixing between locations and may inform parameter estimates that are difficult to make using surveillance data alone (Pybus et al., 2012).

In the near future, we anticipate that progress towards phylodynamic studies of other pathogens will follow sequencing technology. Existing frameworks designed for HIV, hepatitis C and influenza A can be adapted to other RNA viruses, such as common acute respiratory viruses (e.g. respiratory syncytial virus) and enteric viruses (e.g. norovirus, rotavirus), for which sequence data are being generated as part of vaccine studies. DNA viruses with large genomes, which employ very different mechanisms to avoid and subvert host immunity, are also amenable to phylodynamic studies (see e.g. Kerr et al. (2012)), and there is great potential for studying the transmission of common herpesviruses such as cytomegalovirus and Epstein-Barr virus. Many individuals carry – but do not suffer infection from – potentially pathogenic bacteria such as *Staphylococcus aureus* and *Streptococcus pyogenes*, and phylodynamic approaches may provide insights into the spatiotemporal patterns of transmission, carriage and disease. In the longer term, we anticipate more phylodynamic studies on eukaryotic pathogens, such as fungi (e.g. *Candida albicans*) and protozoans, such as malaria. As research expands into more host-pathogen systems, many more challenges than those we have outlined are likely to present themselves.
